# Viral nervous necrosis in gilthead sea bream (*Sparus aurata*) caused by reassortant betanodavirus RGNNV/SJNNV: an emerging threat for Mediterranean aquaculture

**DOI:** 10.1038/srep46755

**Published:** 2017-05-02

**Authors:** Anna Toffan, Francesco Pascoli, Tobia Pretto, Valentina Panzarin, Miriam Abbadi, Alessandra Buratin, Rosita Quartesan, Daniel Gijón, Francesc Padrós

**Affiliations:** 1 OIE reference centre for viral encephalopathy and retinopathy, Istituto Zooprofilattico Sperimentale delle Venezie, Viale dell’Università 10, Legnaro, Padova, Italy; 2 Istituto Zooprofilattico Sperimentale delle Venezie, Fish Pathology Department, Via Leonardo da Vinci 39, Adria, Rovigo, Italy; 3 Department of Veterinary Medical Sciences, Alma Mater Studiorum University of Bologna, Via Tolara di Sopra 50, Ozzano dell’Emilia, Bologna, Italy; 4 Fish Health Service, Skretting, Ctra. de la Estación S/N, Cojóbar, Spain; 5 Fish Diseases Diagnostic Service, Facultat de Veterinaria, Universitat Autònoma de Barcelona, Bellaterra, Cerdanyola del Vallès, Spain.

## Abstract

Viral nervous necrosis (VNN) certainly represents the biggest challenge for the sustainability and the development of aquaculture. A large number of economically relevant fish species have proven to be susceptible to the disease. Conversely, gilthead sea bream has generally been considered resistant to VNN, although it has been possible to isolate the virus from apparently healthy sea bream and sporadically from affected larvae and postlarvae. Unexpectedly, in 2014–2016 an increasing number of hatcheries in Europe have experienced mass mortalities in sea bream larvae. Two clinical outbreaks were monitored over this time span and findings are reported in this paper. Despite showing no specific clinical signs, the affected fish displayed high mortality and histological lesions typical of VNN. Fish tested positive for betanodavirus by different laboratory techniques. The isolates were all genetically characterized as being reassortant strains RGNNV/SJNNV. A genetic characterization of all sea bream betanodaviruses which had been isolated in the past had revealed that the majority of the strains infecting sea bream are actually RGNNV/SJNNV. Taken together, this information strongly suggests that RGNNV/SJNNV betanodavirus possesses a particular tropism to sea bream, which can pose a new and unexpected threat to the Mediterranean aquaculture.

Viral nervous necrosis (VNN), also known as viral encephalopathy and retinopathy (VER), is one of the most devastating infectious diseases affecting marine aquaculture. Since its first description in the late ‘80 s, a large number of economically relevant fish species have proven to be susceptible to the disease, such as European sea bass (*Dicentrarchus labrax*), Asian sea bass (*Lates calcarifer)*, groupers (*Ephinephelus spp*.), Striped jack *(Pseudocaranx dentex*), Senegalese sole (*Solea senegalensis*), turbot (*Scophthalmus maximus*), Atlantic halibut (*Hippoglossus hippoglossus*) and Atlantic cod (*Gadus morhua*)^1^,^2^.

Diseased fish typically show abnormal swimming behavior and nervous signs caused by the viral replication in the cells of brain and retina. Skin color variations, anorexia, lethargy or swim bladder hyperinflation are also frequently observed^2^. VNN is caused by *Betanodavirus,* a naked positive-sense single-stranded RNA virus belonging to the family *Nodaviridae*. The viral genome is made of two genetic segments containing three open reading frames (ORFs). The RNA1 gene of approximately 3.1 kb encodes the viral replicase and the RNA2 segment of ca. 1.4 kb encodes the capsid protein. A third transcript, known as RNA3 (0.4 kb), is cleaved from the RNA1 terminus during viral replication and encodes the B2 non-structural protein, an inhibitor of cell RNA silencing^3^,^4^. To date, four different betanodavirus species have been officially recognized by the International Committee on Taxonomy of Viruses (ICTV), namely the red-spotted grouper- (RGNNV), the striped jack- (SJNNV), the barfin flounder- (BFNNV) and the tiger puffer nervous necrosis virus (TPNNV)^5^. Due to the segmented nature of their genome, fish nodaviruses can undergo genetic shift resulting in the generation of reassortant viruses. To date, the reassortants RGNNV/SJNNV and SJNNV/RGNNV have been described^6^,^7^,^8^, whose genotype name designation refers to the donor genotype of the polymerase/capsid protein genes (RNA1/RNA2, respectively). Betanodaviruses can be classified into three different and partially cross-reactive serotypes: serotype A, which includes SJNNV and RGNNV/SJNNV viruses, serotype B that consists of the BFNNV and the TPNNV genotypes and serotype C, which encompasses RGNNV and SJNNV/RGNNV strains^9^. It is worth mentioning how different viral species show diverse phenotypic features, including temperature dependency, mainly regulated by the RNA1^10^,^11^, as well as species tropism and immunoreactivity, determined by the RNA2^9^,^12^,^13^. As a result, betanodavirus pathogenicity is influenced by both environmental conditions and host species^14^,^15^,^16^,^17^,^18^.

VNN is endemic in the Mediterranean basin and the occurrence of the disease has been widely documented both in farmed and wild fish. The RGNNV is the most commonly detected virus species in this area^2^, although the SJNNV species was also reported in the Iberian Peninsula in 2009^8^. Notably, the occurrence of the reassortant strains RGNNV/SJNNV and SJNNV/RGNNV has been reported only in the Mediterranean Sea^19^.

Gilthead sea bream (*Sparus aurata*), with a production of 146.467 tons in 2014 (source: FEAP), together with European sea bass (*Dicentrarchus labrax*) are the two most relevant species reared in the Mediterranean basin. Sea bream has generally been considered resistant to VNN as no mortality or disease signs are usually detected in routine rearing conditions. Indeed, this species has often been reared in close vicinity to sea bass in areas where VNN is endemic. Interestingly, most of the VNN outbreaks in seabass were associated to the RGNNV species^20^,^21^,^22^, which suggested that maybe sea bream was not susceptible to this specific virus species. This finding was supported also under experimental conditions. As a matter of fact, RGNNV-challenged sea bream showed mortalities only in juveniles and only if infected by intramuscular route, while older fish suffered mild or no clinical signs nor mortality despite testing positive for betanodavirus^23^,^24^,^25^. A role of sea bream as an asymptomatic contagious host for sea bass was therefore postulated. Indeed, only few sporadic and occasional field mortality events associated to betanodavirus in sea bream have been reported in the past years^26^,^27^,^28^, which may account for the limited knowledge on VNN pathogenesis and epidemiology in this species.

Unexpectedly, in the last two years an increasing number of marine hatcheries and nurseries in the Mediterranean Sea have experienced severe disease outbreaks and high mortality in sea bream larvae, postlarvae and juveniles caused by betanodavirus infection. The aim of this manuscript is to provide evidence to the scientific community of the threat posed by VNN to sea bream, a species that up to now was considered resistant to the disease. To reach this goal: (i) field and laboratory findings from two disease outbreaks were described; (ii) VNN isolates were genetically characterized and their molecular epidemiology investigated.

## Results

### Case history and sampling

Two outbreaks in two different fish farms were monitored over a 1-year period. The farms were located in two different European countries with no epidemiological connections. Both farms agreed to provide samples as well as clinical and epidemiological data, but under a confidentiality agreement, which explains why information on the geographic location will not be provided.

#### Farm 1

In September 2014 a new batch of 250 Gilthead sea bream broodstock was introduced into farm 1, which had had no recent history of VER. Fish were divided into family groups of 50 specimens each, and after a short period of acclimation they were introduced into the hatchery for reproduction. The hatched larvae were bred in circular tanks with marine well water (filtered well water, salinity 37‰, temperature 19 °C, density 150/180 larvae per litre) and fed with in-house produced rotifers. Unexpectedly, the newly hatched larvae started dying. Initially, mortality was attributed to technical problems, but due to its persistence a disease was suspected. Affected larvae showed severe lethargic behaviour and apathy, anorexia and finally died. Uncoordinated swimming and/or swimbladder hyperinflation was observed only in a small number of specimens. In some tanks, mortality started at 17–20 days post hatch (dph), while in other tanks it started at 20–30 dph. The sooner the larvae were affected, the higher the mortality was. Cumulative mortality was dramatic, ranging from 80 to 98%.

Pools of diseased larvae from 10 to 70 dph were collected and sent to the Istituto Zooprofilattico Sperimentale delle Venezie (IZSVe) laboratory for diagnostic investigation, which confirmed the presence of betanodavirus. Additionally, fresh larvae were fixed in 10% buffered formalin and sent along with refrigerated fish to the laboratory.

Larvae that survived over 50–70 dph, slowly started to recover and after two months started to adjust to normal feeding and growing patterns. A group of survivor fish from the acute phase of the infection was reared in an outdoor concrete tank (19 °C) for almost 10 months (290 dph). The recovered fish showed to be particularly susceptible to bacterial infections and were repeatedly treated with antibiotics. From these fish, pools of 10 brains each were sent every month to the IZSVe laboratory in order to regularly check the betanodavirus persistence. In addition, at 219 dph brains were individually collected from 22 fish and tested. Serum samples (*n* = 22) were also collected from 290 dph fish and tested individually for the presence of antibodies against betanodaviruses.

In order to identify the origin of the infection, 41 out 250 specimens from the initial broodstock (12 females, 9 males and 20 undetermined) were randomly sacrificed and brain, eye and gonads samples were collected and tested individually by real-time RT-PCR (rRT-PCR) and virus isolation analysis. Additionally, 114 serum samples were randomly collected from the remaining breeders to be tested. Notably, broodstock never showed any clinical sign referable to VNN or any other disease.

Artemia and rotifers were also routinely checked by rRT-PCR for nodavirus presence. In July 2015 the fish farm owner decided to sacrifice and destroy the infected sea bream larvae/juveniles and the remaining broodstock in order to clean, disinfect and fallow the whole hatchery.

#### Farm 2

In October 2015, an increase in mortality of unknown origin occurred in a hatchery with no previous VNN history. New fish were not introduced in the facilities as a general policy. Initially, mortality in larvae was associated to a previously occurred pump failure, with the consequent increase in water turbidity and drop in temperature for some hours. Larvae were housed at a density of 125 larvae per litre in filtered and UV sterilized sea water at 34–35‰ salinity and at an average temperature of 19 °C. The first mortality event reached a high percentage (60%) and affected only 50–70 dph postlarvae, although it was assumed it might have been directly associated to a technical problem. The water was extremely turbid and a treatment with florfenicol to prevent tenacibaculosis outbreaks was immediately implemented. Six days later mortality dropped, although it started to rise again after 3 days (i.e., at day 9 of treatment). Clinical signs, such as apathy and nervous behaviour when stimulated were noticed in a few subjects only and seemed to be similar to those identified in sea bass affected by VNN, although not as evident. Whole larvae, frozen and kept in RNALater™, were thus collected, sent to the IZSVe laboratory. Betanodavirus was detected through virological and biomolecular techniques. Fixed larvae in buffered 10% formalin were also sent to the Universitat Autònoma de Barcelona (UAB) for histopathological analysis. The surviving fish (60–80 dph) were transferred to a separate facility with water recirculation and divided into 6 tanks with disinfection of inlet and outlet water at a temperature of 19 °C. A few weeks after the clinical phase of the disease, mortality progressively disappeared and survivor fish recovered completely. From these fish, pools of brain were collected and tested weekly by rRT-PCR during the following 7 months. Brain (n = 60) and serum samples (n = 31) were also collected at 150 dph and tested individually. Samples for histopathology from apparently normal fish and from some others displaying altered swimming behaviour were also sent to the UAB. In the rest of the farm, protocols of isolation, sanitization and disinfection were applied. Artemia and rotifers where also checked for nodavirus presence by rRT-PCR.

In order to investigate the epidemiology of the disease, 85 breeders of the affected postlarvae were sacrificed and samples of brain and gonads were obtained for rRT-PCR. All the fish had been in the farm for a long period of time and appeared to be healthy. One hundred and seventy blood samples were also randomly collected from the remaining breeders and serum was obtained for antibodies detection. The larval and postlarval facilities were isolated, cleaned, disinfected and fallowed for a month.

Production finally restarted, although in January 2016 a new outbreak of VNN appeared in several tanks of sea bream larvae at 25–35 dph. Samples of the larvae were immediately sent to both laboratories and VNN was confirmed after a few days. In light of what had happened during the previous outbreak, the fish farm owner decided to sacrifice and destroy the whole larvae batch and stop the production in order to prevent the spreading of the pathogen. The survivor fish in separate recirculation units where maintained in this facility until May 2016, when it was decided to remove them and adequately destroy the carcasses. Between February and May 2016, in one of the tanks in the recirculation system, survivor fish were kept in cohabitation with 80–100 g sea bream and sea bass from previous batches. No signs of disease were recorded in these animals. These fish were regularly sampled and brains were sent to the IZSVe for betanodavirus detection.

### Laboratory analysis

In both farms, parasitological and bacteriological tests were performed to detect common parasites/bacteria pathogenic for sea bream; however, the outcome was either negative or leading to the identification of ubiquitous bacteria.

Artemia, rotifers and broodstock samples (brain, eyes and gonads) from both farms always tested negative to rRT-PCR; in addition, broodstock samples turned out to be negative also to virus isolation.

Pools of diseased sea bream larvae/heads from both farms always yielded positive results by rRT-PCR with both protocols (targeting the RNA2 and the RNA1). Virus isolation, when applicable, always confirmed biomolecular results.

Recovered sea bream from both farms continuously tested positive. In farm 1 the fish tested monthly by rRT-PCR were positive up to 219 dph. Notably, at 219 dph 22 out of 25 brain samples tested positive. The virus could still be easily isolated in 18 of the 22 rRT-PCR positive samples. In farm 2, the recovered fish sampled on a weekly basis generally tested positive by rRT-PCR, although some samples turned out to be negative after 4–5 months from the beginning of the outbreak. However, among the specimens which had tested positive by rRT-PCR at 150 dph, virus isolation was still possible in 11 samples out of 12 (11/12).

**Figure 1:**
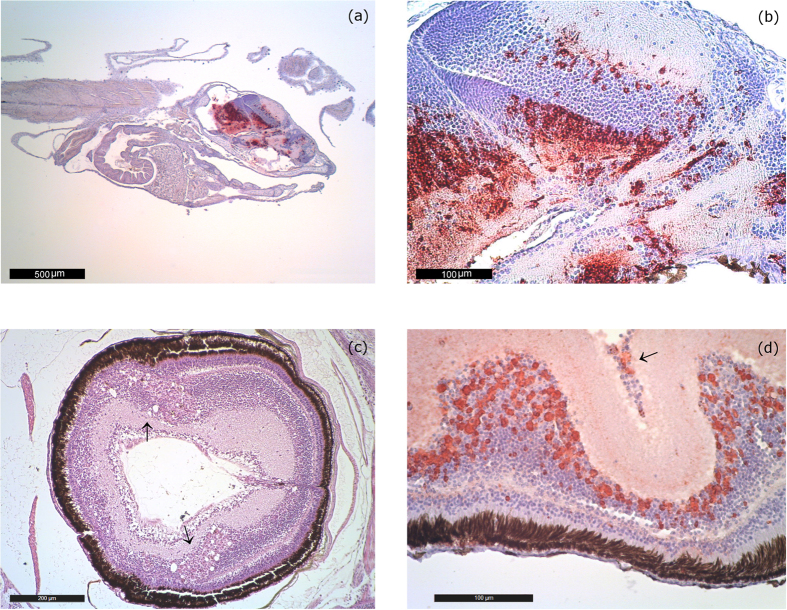
Acute lesions. (**a**) IHC Sea bream larvae of 16 days of age from farm 1 (2014–15). Bright red immunoprecipitates are visible in the telencephalon, mesencephalon, diencephalon (hypothalamus) and cerebellum. IHC labeling is generally higher in larvae and post larvae than in juveniles. 40 magnification. (**b**) IHC of 16 day-old larvae from farm 1 (2014–15). Massive immunoprecipitates in the telencephalon, mesencephalon, diencephalon (hypothalamus). Remarkably, no vacuolization is noticeable. 250 magnification. (**c**) Sea bream postlarvae of 45 days of age from farm 1 (2014–15). Vacuolation in the inner nuclear layer of the retina (arrow). H&E 100 magnification. (**d**) IHC of a 55 day-old seabream eye collected in farm 1 (2014–15). Immunoprecipitates are evident in the inner nuclear layer and the ganglion cell layer (arrow) of the retina. 250 magnification.

In the most acute cases (larvae and post larvae), histological examination showed moderate VNN-associated lesions (Fig. 1). Vacuolations were present in the telencephalon, mesencephalon and cerebellum. The inner nuclear layer and the ganglion cell layer of the retina also showed vacuolization. Immunohistochemistry (IHC) confirmed the etiology of the visible lesions with the presence of massive immunoprecipitates in the nervous tissues. In many cases, immunoprecipitates were also present in the absence of vacuoles (Fig. 1a,b,d).

**Figure 2:**
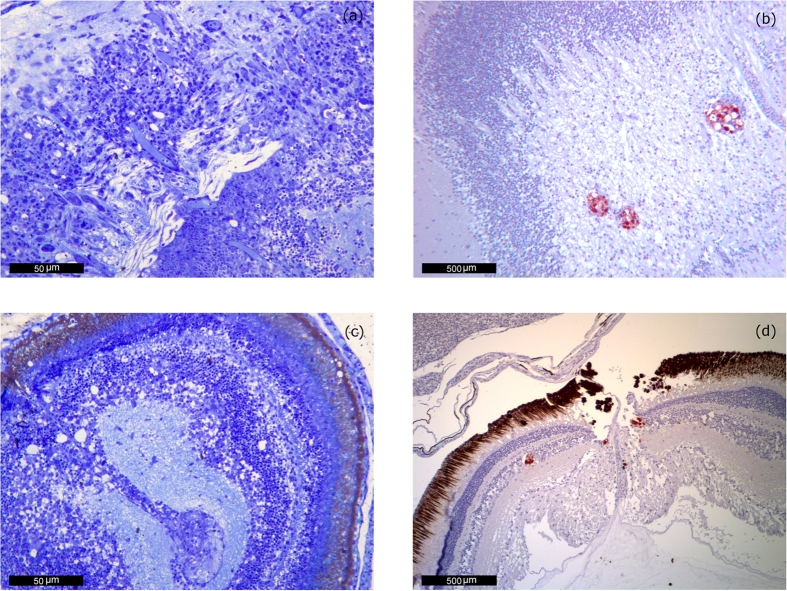
Chronic lesions. (**a**) Sea bream postlarvae, 47 days of age from farm 2 (2015–16). Development of apparently inflammatory response (gliosis) and neuronal necrosis but with little accompanying vacuolization. Methacrylate section. Toluidine Blue stain (TB). 400 magnification. (**b**) IHC of 6-month old juvenile sea bream juvenile from farm 1 (2014–15). Mesencephalon (optic tectum) IHC signal is reduced to some small foci surrounding vacuoles. 100 magnification. (**c**) Eye of a 47 day-old postlarvae from farm 2 (2015–16). Alteration of the inner nuclear layer of the retina. Some cells display necrotic changes but only few of them present cytoplasmic vacuolation. Methacrylate section. Toluidine Blue stain (TB). 400 magnification. (**d**) IHC of 6-month old juvenile sea bream from farm 1 (2014–15). Cluster of immunoprecipitates is visible in the inner nuclear layer next to the emergence of the optic nerve. 100 magnification.

In survivors and juveniles, lesions tended to be more limited and sometimes difficult to observe (Fig. 2). The development of an apparently inflammatory response (gliosis) was indeed typical. Neuronal necrosis was also present, but with a low intensity just as in the acute phase and with few accompanying cytoplasmic vacuolization. In these cases also the IHC signal was usually reduced to some small foci of a red bright color (Fig. 2b,d), while the areas with the most intense inflammatory reaction did not display any labeling.

**Figure 3:**
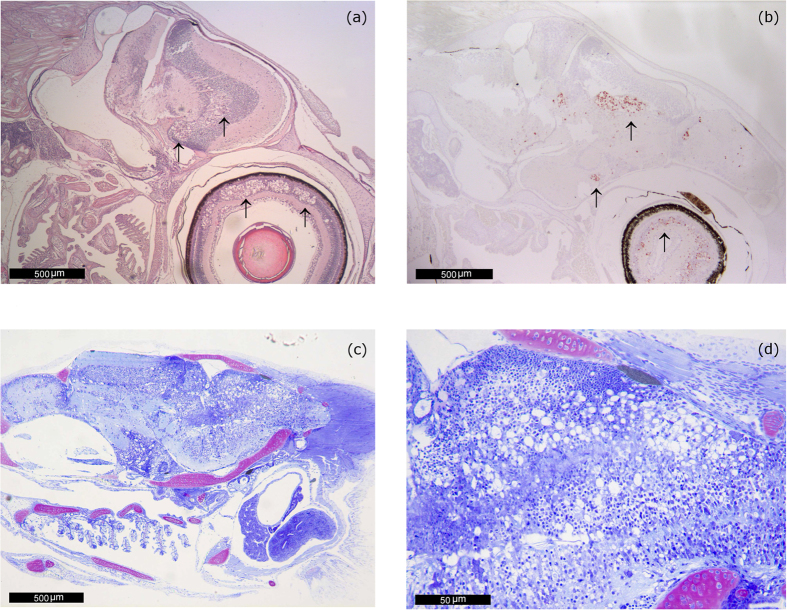
Lesions in past VER outbreaks in sea bream. (**a**) VNN-associated damage in the encephalon and in the retina (arrows) of a 45 day-old seabream from samples collected during the Cyprus outbreak in 2010. H&E. 40 magnification. (**b**) IHC of a 45 day-old seabream brain from another specimen collected during the Cyprus outbreak (2010). Immunoprecipitates are visible in the telencephalon (olfactory lobes, preoptic nucleus) in mesencephalon (tectum opticum and tegmentum) and retina (arrows). 40 magnification. (**c**) 43 day-old Sea bream postlarvae collected during the 2005 outbreak in Portugal. Acute, severe and extensive lesions. Extensive vacuolation is noticeable in the telencephalon, mesencephalon, diencephalon (hypothalamus) and medulla oblongata. Methacrylate section. Toluidine Blue stain (TB). 40 magnification. (**d**) 43 day-old Sea bream postlarvae collected during the 2005 outbreak in Portugal. Higher magnification of the lesions observed in (**c**). Notice the presence of a large number of cells with large vacuoles within the cytoplasm in the metencephalon and also in the mesencephalon. Methacrylate section. Toluidine Blue stain (TB). 400 magnification.

In stored paraffin-embedded organs obtained from past VER outbreaks in sea bream, which were retrieved and re-analyzed, the lesions were even more severe than those observed in farms 1 and 2 (Fig. 3). Extensive vacuolation was present in the brain (telencephalon, mesencephalon, cerebellum, diencephalon, hypothalamus, medulla oblongata) and in the retina (inner nuclear layer and ganglion cell layer).

Results of serological analysis showed that only few (7/114) sera from breeders in farm 1 tested slightly positive by serum neutralization (SN) (performed against SJNNV antigen) but negative by immunofluorescence (IF) (Table 1). All sera collected from broodstock in farm 2 (n = 170) tested negative by SN.

Sera (n = 53, 22 from farm 1 and 31 from farm 2) collected from recovered, but still positive at virological analysis, sea bream, tested negative by SN and IF. Serological tests were standardized by including in each plate a positive sea bream sera produced by immunizing adult sea bream with SJNNV virus (484.2009 strain^11^). The control sera produced clearly detectable signals in SN and IF (Table 1).

**Figure 4:**
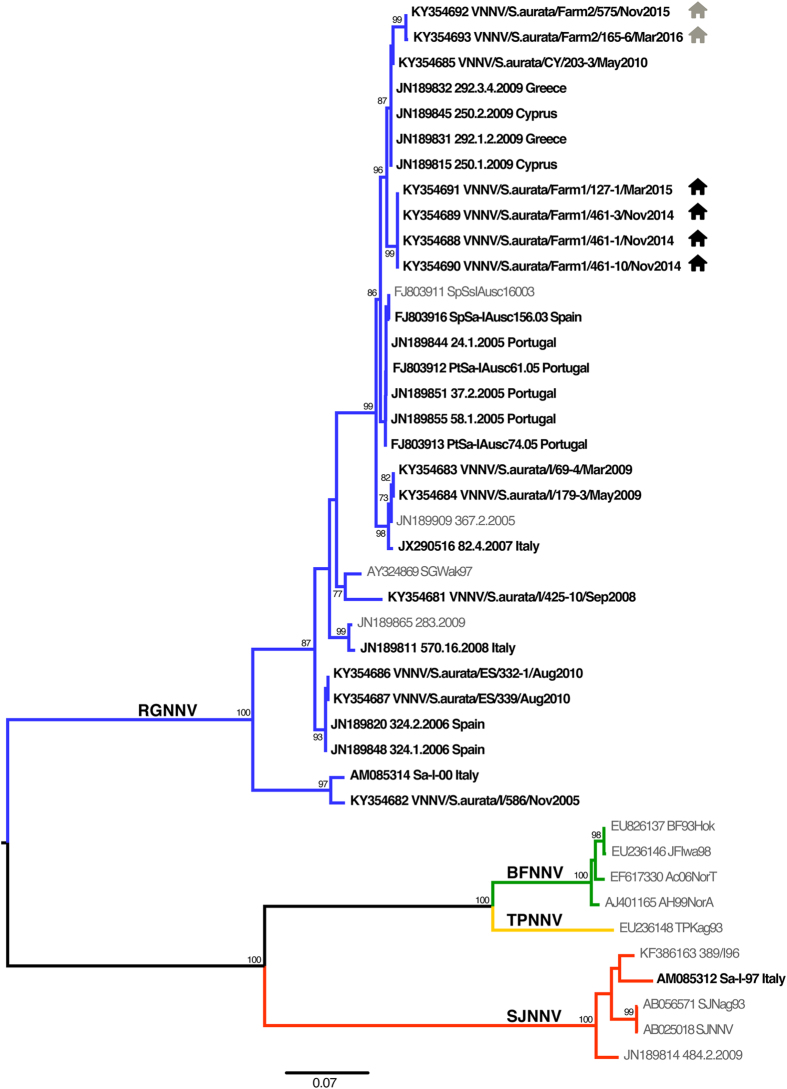
RNA1 Phylogenetic tree. ML phylogenetic tree based on partial RNA1 sequences. Sea bream betanodaviruses are highlighted in bold and the country of origin is reported. The farms of provenance of the strains herein described are labeled as follows: 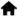 farm 1; 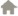 farm 2. Betanodavirus genotype subdivision is displayed by labeling the branches with different colors (blue: RGNNV; green: BFNNV; yellow: TPNNV; red: SJNNV). The numbers at the nodes represent bootstrap values (only values >70% are reported), while branch lengths are scaled according to the number of nucleotide substitutions per site. The scale bar is reported.

**Figure 5:**
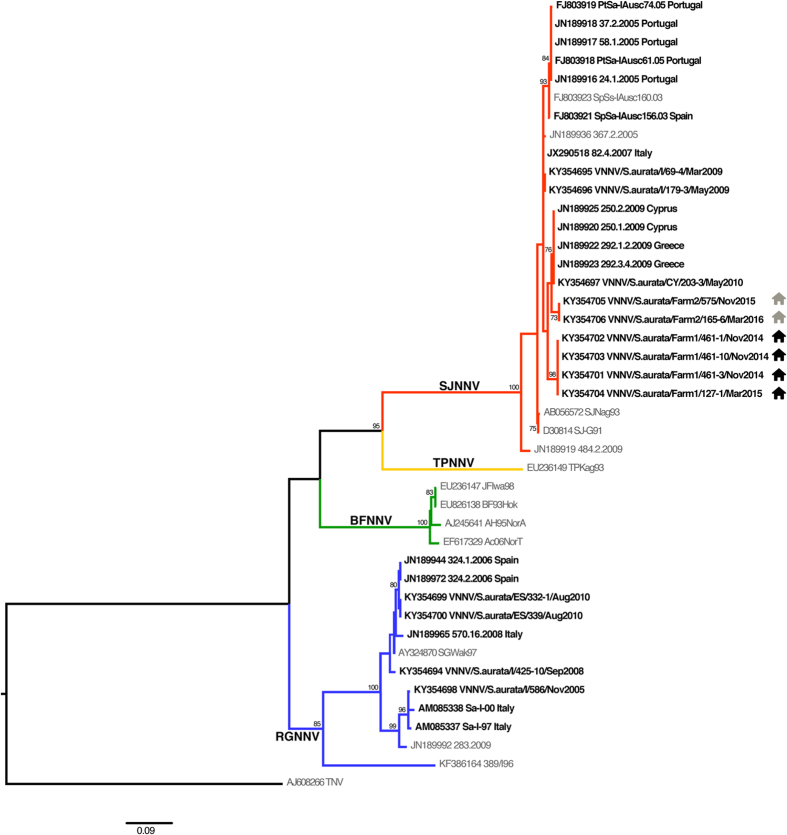
RNA2 Phylogenetic tree. ML phylogenetic tree based on partial RNA2 sequences. Sea bream betanodaviruses are highlighted in bold and the country of origin is reported. The farms of provenance of the strains herein described are labeled as follows: 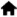 farm 1; 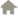 farm 2. Betanodavirus genotype subdivision is displayed by labeling the branches with different colors (blue: RGNNV; green: BFNNV; yellow: TPNNV; red: SJNNV). The numbers at the nodes represent bootstrap values (only values >70% are reported), while branch lengths are scaled according to the number of nucleotide substitutions per site. The scale bar is reported.

The phylogenetic analysis showed that all the isolates from farms 1 and 2 subjected to molecular characterization (n = 6) were reassortant strains RGNNV/SJNNV, showing a RGNNV-type RNA1 and a SJNNV-type RNA2 (Figs 4 and 5). The RNA1 and RNA2 sequences of the sea bream betanodaviruses retrieved from GenBank or obtained from viral isolates stored at the IZSVe repository comprise 8 RGNNV strains from Italy and Spain, 14 RGNNV/SJNNV viruses from Cyprus, Greece, Italy, Portugal and Spain and 1 SJNNV/RGNNV reassortant strain from Italy (Table 2). An RNA2 phylogenetic tree, which includes sea bream betanodaviruses for which the RNA1 sequence is not available or spans a different region of the polymerase gene, is provided as Supplementary material. Of these strains, 13 samples from Portugal are reported to be RGNNV/SJNNV^6^ and 3 viruses from Tunisia are described as being RGNNV^20^. Nucleotide similarities among the RNA1 sequences of RGNNV sea bream betanodaviruses range between 91.4 and 100%, while those reported among the RGNNV/SJNNV viruses span between 97.7 and 100%. RNA2 nucleotide identities among RGNNV and RGNNV/SJNNV are 93–100% and 97–100%, respectively. At the amino acid (aa) positions 247 and 270, all the betanodavirus strains from sea bream present a serine (Ser_247_ and Ser_270_), with the exception of strains Sa-I-97, Sa-I-00 and VNNV/S.aurata/I/586/2005, which show an asparagine at position 270 (Asn_270_).

## Discussion

Betanodaviruses have emerged as a major constraint to marine aquaculture and are responsible for serious losses reported worldwide. They are geographically widespread, show a broad host range, cause severe disease, and no treatment is currently available. Some promising vaccines are presently under study, although to date none are available in Europe^29^.

Fish nodaviruses can infect a broad variety of species^2^ belonging to 30 different families; however, Sparids, in particular Gilthead sea bream, have generally proved to have a low susceptibility or to be resistant to the disease^23^,^24^,^30^. This is probably true when referring to VNN caused by the RGNNV genotype, the most common pathogenic strain for European sea bass. However, the increasing number of reports of RGNNV/SJNNV-related disease in *S. aurata* documented in recent years suggests that this type of reassortant might represent an emerging problem for Mediterranean sea bream farming. Notably, the same reassortant strain has recently proved to possess a peculiar tropism and a high pathogenicity also for flatfish^16^,^31^.

The retrospective analysis on stocked viruses and the plain association between the presence of the reassortant and clinical signs/mortality further confirm this suspicion. In this paper, field and laboratory findings of clinical VNN caused by reassortant betanodavirus RGNNV/SJNNV in *S. aurata* are exhaustively described for the first time, finally demonstrating the susceptibility of this species to the disease. Additionally, results point out that the reassortant RGNNV/SJNNV shows high virulence and specific tropism for *S. aurata* mainly for larvae and postlarvae. Indeed, European sea bass were present in both farms where the VNN outbreaks had occurred, and no evident clinical signs were reported in this species even after cohabitation with RGNNV/SJNNV carrier fish.

The phylogenetic analyses of viruses from farms 1 and 2, of strains inventoried at the IZSVe repository and of sequences retrieved from GenBank highlight that RGNNV/SJNNV is the most frequently detected species in farmed sea bream, which could explain the favourable adaptation of this virus to *S. aurata*. However, it must be mentioned that RGNNV and SJNNV/RGNNV strains have also been sporadically detected, although no data on virus characterization are available with reference to these latter. The few betanodavirus outbreaks in sea bream described in the past^26^,^27^ had very likely been caused by reassortant betanodavirus, as recorded in the only report in which a complete viral characterization is described^28^. Interestingly, Souto and colleagues^15^,^31^ identified two putative molecular determinants for RGNNV/SJNNV at the aa positions 247 and 270 (Ser_247_; Ser_270_), which were found to be at the base of the virulence for Senegalese sole and turbot. It is worth mentioning that all but three sea bream betanodavirus characterized in the present study display the same residues. The role of these mutations in the adaptation and virulence of RGNNV/SJNNV for sea bream clearly requires additional investigations. Notably, all the viruses characterized in the present study showed a marked genetic similarity, despite the time of collection and the country of origin. This observation is consistent with a disease spread associated with anthropogenic activities, during which viruses can be transmitted through different farms trading, transport and management practices^32^.

In the two farms here described, several investigations were performed in order to understand the origin of the infection. In farm 1 the occurrence of the virus was correlated with the introduction of new broodstock. A vertical transmission of the disease at the beginning of the outbreak was therefore postulated. Indeed, this transmission route is well established for betanodavirus and it has been described also in European sea bass and in grouper^33^,^34^,^35^,^36^. The vertical transmission hypothesis was confirmed by the extremely young age of some batches of infected sea bream larvae (10–15 days post hatchery). On the other hand and possibly in a second moment, horizontal transmission from tank to tank probably played a major role in spreading the disease throughout the hatchery, facilitated by the high viral load of the infected larvae (data not shown) and the insufficient application of biosecurity measures.

In farm 2, no recent introduction of new animals had been reported prior to the outbreak and the disease appeared in larvae (50–70 dph) older than those in farm 1. The origin of the virus was therefore linked to a failure in the inlet sea water UV disinfection system. In this case, the horizontal transmission inside the hatchery substantially contributed to the spread of the infection. The environmental contamination by the virus and the posterior redistribution in the facilities by water supply due to inadequate filtration and UV disinfection should also be considered. Finally, the presence of potential carriers or vectors of the virus amongst wild fish species or other organisms living in the aquatic environment where the water supply for the hatchery is located could not be ruled out. Betanodavirus presence was in fact reported in several wild species^37^.

In order to confirm or exclude vertical transmission, organs and sera from a great number of breeders coming from both farms were tested. Organs always resulted negative, which suggests that either broodstock was not responsible for the dissemination of the virus or that the prevalence of infection was particularly low. The development of reliable (and possibly non-invasive) tests for determining the virus-free status of broodstock will be a critical issue for the future.

From an epidemiological point of view, the fact that sea bream survivors tested positive to the virus (both by rRT-PCR and virus isolation) for months after the infection is extremely relevant. The virus could either reactivate under stressful conditions (i.e., transportation, rough management) or, alternatively, it could be transmitted to susceptible fish. In any case, introducing sea bream that look perfectly healthy into a farm without any laboratory confirmation of their noda-free status could pose a serious threat.

With reference to serological results, samples tested by SN yielded principally negative or weakly positive results of uncertain interpretation. The arrangement of experimental infections in order to investigate the level and duration of seroconversion in recovered sea bream as well as the development and validation of serological tests (in particular ELISA) will help to correctly interpret serological results.

In 2007 an outbreak of betanodavirus in sea bream of a weight between 50–80 g farmed in sea cages in Greece^27^ was reported. At that time the viral isolate was not genetically characterized by the authors but it may very likely have been a RGNNV/SJNNV. Barring this exception, to date almost all VNN outbreaks in sea bream have occurred only in hatcheries and nurseries. What is still unclear is whether juveniles/adult fish are as susceptible as larvae or if they are likely to be more resistant. In the cohabitation experiences performed in farm 2, virus- positive survivors were unable to transfer the virus to bigger sea bream and to sea bass. Indeed, the expected resistance of this latter species to the reassortant RGNNV/SJNNV may account for the lack of transmission of the disease^16^,^17^. However, for the bigger cohabitating sea bream, this could be due to the higher resistance of elder fish or to the reduced infectivity of the survivors after the acute phase. Signals detected by rRT-PCR as well as viral labelling by IHC were much lower in survivor fish/chronic stages of the disease than in symptomatic ones.

In conclusion, sea bream is an important species in terms of economic value and has frequently been used as an alternative species to sea bass in VNN endemic areas, so as to reduce the concentration of susceptible biomass. The spreading of VNN disease to sea bream farms could severely impact on Mediterranean aquaculture. It is therefore of paramount importance to improve the knowledge on VNN pathogenesis in *S. aurata* and to invest resources in epidemiological surveys as well as on the development of prevention strategies.

## Methods

### Bacteriological and parasitological analyses

Bacteriological analyses from CNS and, when available, from kidney and spleen of fingerling/adult sea bream were carried out according to standard procedures and plated on blood agar and marine agar. Pool of larvae were rinsed with sterile saline solution and grinded with sterile sand in a mortar before plating on blood and marine agar. Parasitological tests were performed by microscopic observation of gill and skin according to standard procedures.

### Virus isolation and biomolecular analyses

Virological investigations were performed on different sample matrices: pools of whole bodies for larvae and fish smaller than 0, 5 cm, pools of 10 cut head for fish up to 2 cm, pools of 10 brains for fish bigger than 2 cm, individual organs (brain, eye, gonads) for adult fish. Specimens were sent to the laboratory either frozen or stored in RNA later™. The samples were weighed, manually homogenized with pre-dosed sterile quartz sand and diluted 1:3 with medium (MEM, Sigma-Aldricht). Samples were then centrifuged for 15 minutes at 4000 × *g* in a refrigerated centrifuge and supernatant harvested and submitted to rRT-PCR and/or virus isolation. RNA was extracted using the NucleoSpin^®^ RNA Kit (Macherey-Nagel) following the manufacturer’s instructions. Two rRT-PCR targeting respectively RNA2 and RNA1^38^,^39^ were used in parallel for most of the samples. Virus isolation and virus titration was performed on SNN-1 cells^40^ according to standard procedures^11^ on rRT-PCR positive samples.

### Histological and immunohistochemistry examination

Brain/eye samples of sea bream whole larvae or juveniles were fixed in 10% buffered formalin and processed for histopathological (paraffin and methacrylate) and immunohistochemical examination according to standard techniques^41^,^42^. For IHC, in-house produced rabbit polyclonal antiserum anti SJNNV^9^ diluted 1:3000 was used.

### Serological analysis

Blood samples were refrigerated for 1 night at 4 °C after collection to allow cloth formation. Serum was harvested and inactivated at 37 °C for 1 h.

#### Serum neutralization

SN was performed using the SJNNV (strain 484.2.2009) as an antigen^9^. Sera were considered as positive when showing neutralizing titres ≥1:40. SN test has been previously validated in our laboratory and has been chosen because this assay is not species-dependent.

#### Immunofluorescence test

E-11 cells^43^ seeded on 96 wells plates were infected with betanodavirus strain 484.2.2009 (SJNNV). Approximately 3 days post incubation at 25 °C, immediately after the appearance of the cytopathic effect, the cell monolayer was fixed with 80% acetone for 1 hour and then washed with phosphate-buffered saline solution (PBS). Sea bream serum was added at different dilutions (1:10–1:50–1:100) and incubated at 37 °C for 90 minutes. After washing, a secondary MAb anti-sea bream IgM (Aquatic Diagnostic Ltd) diluted 1:10 was added and incubated at 37 °C for 90 min. After PBS washing, a fluorescein-conjugated MAb against mouse IgG (Sigma-Aldricht) diluted 1:100 was added and incubated for further 90 minutes. Finally, plates were washed with PBS and immediately read under a fluorescent microscope at 20X–40X.

#### Reference positive sample

Positive sea bream anti-SJNNV serum was produced at the IZSVe animal facilities. Briefly, n = 10 healthy sea bream (weight 180–200 gr) were anesthetized with MS-222 and injected twice (intraperitoneally and then 3 weeks after being boostered by intramuscularl injection) with 100 μl of formalin inactivated betanodavirus. Blood samples collected 30 days after the second inoculum were pooled, the serum was harvested and inactivated at 37 °C for 1 h. This sample was included as positive control in every test plate.

### Genotyping and phylogenetic analysis

A representative number of isolates per farm (Table 2) was subjected to RT-PCR and sequencing performed according to the procedure previously described^44^. For comparison, all the sea bream isolates stored at −80 °C or freeze-dried in the IZSVe repository were also sequenced. Sequence data were assembled and edited using the SeqScape software v2.5 (Applied Biosystems). For completeness, RNA1 and RNA2 sequences related to viral strains detected in sea bream and publicly available in GenBank were included in the phylogenetic analysis, as well.

The RNA1 and RNA2 partial sequences were aligned and compared to representative sequences publicly available in GenBank using the MEGA6 software^45^. To genetically characterize the sea bream isolates, phylogenetic trees based on both genetic segments were inferred using the maximum likelihood (ML) method available in the PhyML program version 3.1^46^. The trees incorporate a general time-reversible (GTR) model of nucleotide substitution with a gamma-distribution of among-site rate variation (with four rate categories, Γ4) and a SPR branch-swapping search procedure^47^. To assess the robustness of nodes, 1000 bootstrap replicates were performed using the GTR model. Phylogenetic trees were visualized with the FigTree v1.4 software (http://tree.bio.ed.ac.uk/software/ figtree/). Pairwise nucleotide similarities (*p*-distance method) among the sea bream betanodavirus sequences were determined with the MEGA6 software^44^.

### Ethical statement

All animal experiments were performed in accordance with relevant guidelines and regulations (Italian law Decree D. Lgs n. 26 on 4^th^ March 2014 and the EU Directive 2010/63/EU). The fish immunization procedure was approved by the Ethics Committee of the Istituto Zooprofilattico Sperimentale delle Venezie (IZSVe) (decision n° CE.IZSVE.05/2012 of the 01^st^ February 2012) and communicated to the Italian Ministry of Health (IZSVe protocol n° 0001797/2012 of the 23^th^ February 2012).

## Additional Information

**How to cite this article:** Toffan, A. *et al*. Viral nervous necrosis in gilthead sea bream (*Sparus aurata*) caused by reassortant betanodavirus RGNNV/SJNNV: an emerging threat for Mediterranean aquaculture. *Sci. Rep.* **7**, 46755; doi: 10.1038/srep46755 (2017).

**Publisher's note:** Springer Nature remains neutral with regard to jurisdictional claims in published maps and institutional affiliations.

## Supplementary Material

Supplementary Figure S1 and Table S2

## Figures and Tables

**Table 1:** Serological results.

Origin	Fish age	ID serum	SN titre	IF
Positive reference serum SJNNV	Adult	24/ITT12 n. 14	1:320	1:100
Farm 1	Broodstock	14ITT/464.3	1:80	Negative
Farm 1	Broodstock	14ITT/464.5	1:40	Negative
Farm 1	Broodstock	14ITT/464.8	1:80	Negative
Farm 1	Broodstock	14ITT/492.8	1:40	Negative
Farm 1	Broodstock	15ITT/42.18	1:80	Negative
Farm 1	Broodstock	15ITT/46.19	1:40	Negative
Farm 1	Broodstock	15ITT/46.45	1:40	Negative

Titers (expressed as limit dilution) of broodstock sea bream sera samples from farm 1were evaluated with serum neutralization (SN) and Immunofluorescence (IF).

**Table 2:** List of sea bream betanodavirus isolates included in the phylogenetic analysis.

ID isolate	Year	Age	Fish status	Origin	Clinical signs	GenBank Acc. No.		Genotype	Reference
						RNA1	RNA2		
VNNV/S.aurata/Farm1/461-1/Nov2014^a^	2014	Larvae	Farmed	Farm 1	Present	KY354688	KY354702	RGNNV/SJNNV	This work
VNNV/S.aurata/Farm1/461-10/Nov2014^a^	2014	Larvae	Farmed	Farm 1	Present	KY354690	KY354703	RGNNV/SJNNV	This work
VNNV/S.aurata/Farm1/461-3/Nov2014^a^	2014	Larvae	Farmed	Farm 1	Present	KY354689	KY354701	RGNNV/SJNNV	This work
VNNV/S.aurata/Farm1/127-1/Mar2015^a^	2015	Larvae	Farmed	Farm 1	Present	KY354691	KY354704	RGNNV/SJNNV	This work
VNNV/S.aurata/Farm2/575/Nov2015^a^	2015	Larvae	Farmed	Farm 2	Present	KY354692	KY354705	RGNNV/SJNNV	This work
VNNV/S.aurata/Farm2/165-6/Mar2016^a^	2016	Larvae	Farmed	Farm 2	Present	KY354693	KY354706	RGNNV/SJNNV	This work
VNNV/S.aurata/I/586/Nov2005^b^	2005	n.a.	EFS	Italy	Absent	KY354682	KY354698	RGNNV	This work
VNNV/S.aurata/I/425-10/Sep2008^b^	2008	Juvenile	Farmed	Italy	Absent	KY354681	KY354694	RGNNV	This work
VNNV/S.aurata/I/69-4/Mar2009^b^	2009	Larvae	Farmed	Italy	Present	KY354683	KY354695	RGNNV/SJNNV	This work
VNNV/S.aurata/I/179-3/May2009^b^	2009	Larvae	Farmed	Italy	n.a.	KY354684	KY354696	RGNNV/SJNNV	This work
VNNV/S.aurata/CY/203-3/May2010^b^	2010	Larvae	Farmed	Cyprus	Present	KY354685	KY354697	RGNNV/SJNNV	This work
VNNV/S.aurata/ES/332-1/Aug2010^b^	2010	Larvae	Farmed	Spain	Absent	KY354686	KY354699	RGNNV	This work
VNNV/S.aurata/ES/339/Aug2010^b^	2010	Larvae	Farmed	Spain	Absent	KY354687	KY354700	RGNNV	This work
Sa-I-97^c^	1997	n.a.	Farmed	Italy	Absent	AM085312	AM085337	SJNNV/RGNNV	Toffolo *et al*.^7^
Sa-I-00^c^	2000	n.a.	Farmed	Italy	Absent	AM085314	AM085338	RGNNV	Toffolo *et al*.^7^
SpSa-IAusc156.03^c^	2003	Larvae	Farmed	Spain	Present	FJ803916	FJ803921	RGNNV/SJNNV	Olveira *et al*.^6^
PtSa-IAusc61.05^c^	2005	Larvae	Farmed	Portugal	Present	FJ803912	FJ803918	RGNNV/SJNNV	Olveira *et al*.^6^
PtSa-IAusc74.05^c^	2005	Larvae	Farmed	Portugal	Present	FJ803913	FJ803919	RGNNV/SJNNV	Olveira *et al*.^6^
24.1.2005^c^	2005	Larvae	Farmed	Portugal	Present	JN189844	JN189916	RGNNV/SJNNV	Panzarin *et al*.^8^
37.2.2005^c^	2005	Larvae	Farmed	Portugal	Present	JN189851	JN189918	RGNNV/SJNNV	Panzarin *et al*.^8^
58.1.2005^c^	2005	Larvae	Farmed	Portugal	Present	JN189855	JN189917	RGNNV/SJNNV	Panzarin *et al*.^8^
324.1.2006^c^	2006	n.a.	Farmed	Spain	Present	JN189848	JN189944	RGNNV	Panzarin *et al*.^8^
324.2.2006^c^	2006	n.a.	Farmed	Spain	Present	JN189820	JN189972	RGNNV	Panzarin *et al*.^8^
82.4.2007^c^	2007	Larvae	Farmed	Italy	Present	JX290516	JX290518	RGNNV/SJNNV	Vendramin *et al*.^18^
570.16.2008^c^	2008	n.a.	Wild	Italy	Absent	JN189811	JN189965	RGNNV	Panzarin *et al*.^8^
250.1.2009^c^	2009	Larvae	Farmed	Cyprus	Present	JN189815	JN189920	RGNNV/SJNNV	Panzarin *et al*.^8^
250.2.2009^c^	2009	Larvae	Farmed	Cyprus	Present	JN189845	JN189925	RGNNV/SJNNV	Panzarin *et al*.^8^
292.1.2.2009^c^	2009	Larvae	Farmed	Greece	Present	JN189831	JN189922	RGNNV/SJNNV	Panzarin *et al*.^8^
292.3.4.2009^c^	2009	Larvae	Farmed	Greece	Present	JN189832	JN189923	RGNNV/SJNNV	Panzarin *et al*.^8^

The following information is reported: years of isolation, age of the specimen, fish status, country or farm of origin, presence of clinical signs, GenBank accession numbers for RNA1 and RNA2 sequences and reference.

n.a.: not available.

EFS: extensive farming system.

^a^Sea bream betanodavirus sequences obtained from diseased specimens from Farm 1 and Farm 2.

^b^Sea bream betanodavirus sequences obtained from viral isolates stored at IZSVe repository.

^c^Sea bream betanodavirus sequences retrieved from GenBank.
