# Supplementary material for: Viral nervous necrosis in gilthead sea bream (Sparus aurata) caused by reassortant betanodavirus RGNNV/SJNNV: an emerging threat for Mediterranean aquaculture
Source: Sci Rep. 2017 May 2;7:46755. doi: 10.1038/srep46755 (PMC5411978; doi:10.1038/srep46755)
Supplement: Supplementary Figure S1 and Table S2 [file srep46755-s1.pdf]

Viral nervous necrosis in gilthead sea bream (*Sparus aurata*) caused by reassortant betanodavirus RGNNV/SJNNV: an emerging threat for Mediterranean aquaculture

Toffan, Anna; Pascoli, Francesco; Pretto, Tobia, Panzarin, Valentina; Abbadi, Miriam; Buratin, Alessandra, Quartesan, Rosita, Gijón, Daniel and Padrós, Francesc

Supplementing figure (S1): RNA2 Phylogenetic tree. ML phylogenetic tree based on partial RNA2 sequences. Sea bream betanodaviruses are highlighted in bold and the country of origin is reported. The farms of provenance of the strains herein described are labeled as follows: farm 1; farm 2. The tree comprises also RNA2 sequences related to viruses for which the RNA1 sequence is not available or spans a different region of the polymerase gene from that used in the present study [1,2]. Betanodavirus genotype subdivision is displayed by labeling the branches with different colors (blue: RGNNV; green: BFNNV; yellow: TPNNV; red: SJNNV). The numbers at nodes represent bootstrap values (only values >70% are reported), while branch lengths are scaled according to the number of nucleotide substitutions per site. The scale bar is reported.

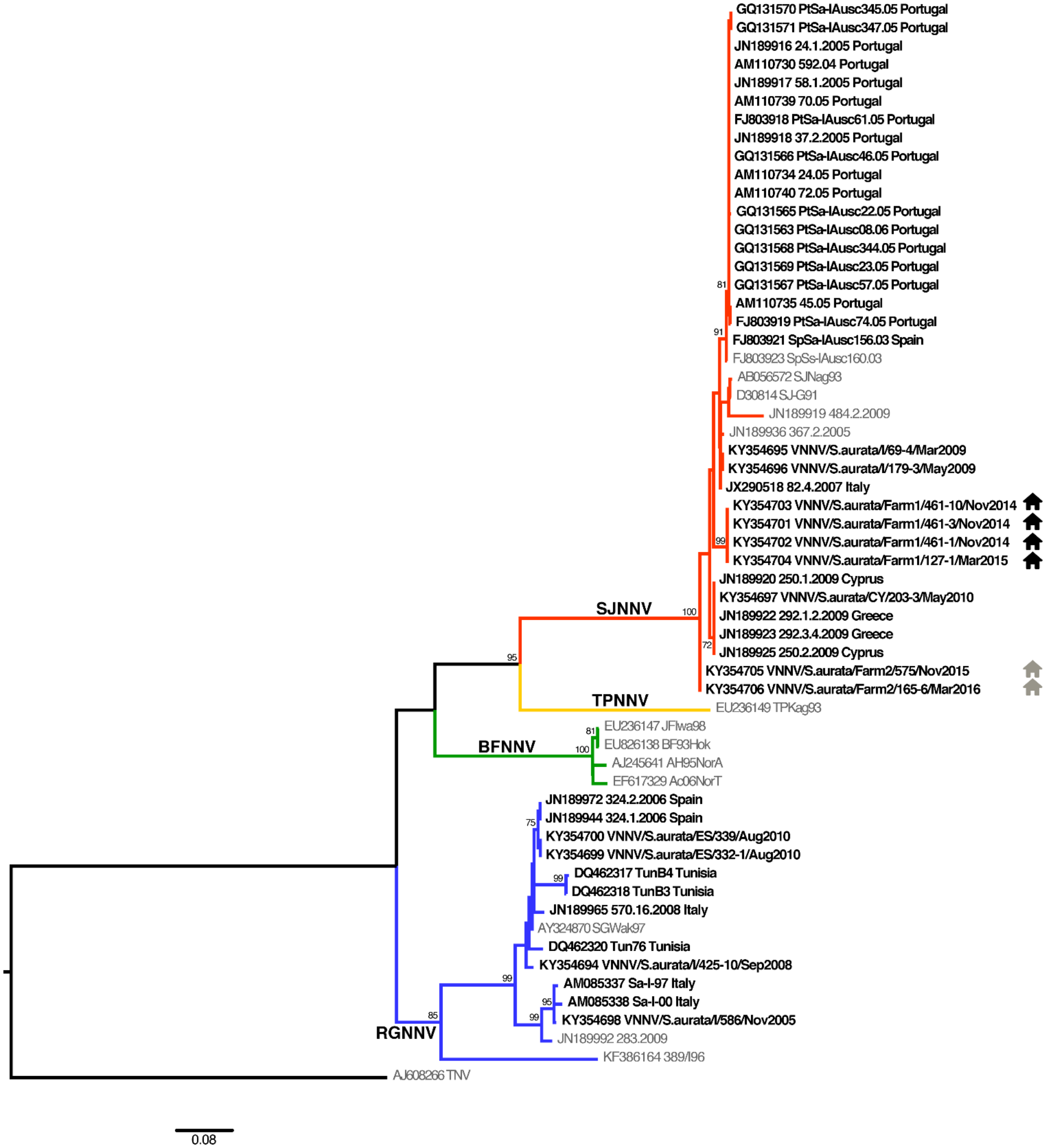

GQ131570 PtSa-IAusc345.05 Portugal
GQ131571 PtSa-IAusc347.05 Portugal
JN189916 24.1.2005 Portugal
AM110730 592.04 Portugal
JN189917 58.1.2005 Portugal
AM110739 70.05 Portugal
FJ803918 PtSa-IAusc61.05 Portugal
JN189918 37.2.2005 Portugal
GQ131566 PtSa-IAusc46.05 Portugal
AM110734 24.05 Portugal
AM110740 72.05 Portugal
GQ131565 PtSa-IAusc22.05 Portugal
GQ131563 PtSa-IAusc08.06 Portugal
GQ131568 PtSa-IAusc344.05 Portugal
GQ131569 PtSa-IAusc23.05 Portugal
81
GQ131567 PtSa-IAusc57.05 Portugal
AM110735 45.05 Portugal
FJ803919 PtSa-IAusc74.05 Portugal
91
FJ803921 SpSa-IAusc156.03 Spain
FJ803923 SpSs-IAusc160.03
AB056572 SJNag93
D30814 SJ-G91
JN189919 484.2.2009
JN189936 367.2.2005
KY354695 VNNV/S.aurata/I/69-4/Mar2009
KY354696 VNNV/S.aurata/I/179-3/May2009
JX290518 82.4.2007 Italy
KY354703 VNNV/S.aurata/Farm1/461-10/Nov2014
KY354701 VNNV/S.aurata/Farm1/461-3/Nov2014
99
KY354702 VNNV/S.aurata/Farm1/461-1/Nov2014
KY354704 VNNV/S.aurata/Farm1/127-1/Mar2015
JN189920 250.1.2009 Cyprus
KY354697 VNNV/S.aurata/CY/203-3/May2010
SJNNV
100
JN189922 292.1.2.2009 Greece
JN189923 292.3.4.2009 Greece
72
JN189925 250.2.2009 Cyprus
95
KY354705 VNNV/S.aurata/Farm2/575/Nov2015
KY354706 VNNV/S.aurata/Farm2/165-6/Mar2016
TPNNV
EU236149 TPKag93
EU236147 JFIwa98
81
BFNNV
EU826138 BF93Hok
100
AJ245641 AH95NorA
EF617329 Ac06NorT
JN189972 324.2.2006 Spain
75
JN189944 324.1.2006 Spain
KY354700 VNNV/S.aurata/ES/339/Aug2010
KY354699 VNNV/S.aurata/ES/332-1/Aug2010
99
DQ462317 TunB4 Tunisia
DQ462318 TunB3 Tunisia
JN189965 570.16.2008 Italy
AY324870 SGWak97
DQ462320 Tun76 Tunisia
KY354694 VNNV/S.aurata/I/425-10/Sep2008
99
AM085337 Sa-I-97 Italy
95
AM085338 Sa-I-00 Italy
85
99
KY354698 VNNV/S.aurata/I/586/Nov2005
RGNNV
JN189992 283.2009
KF386164 389/I96
AJ608266 TNV
0.08

Supplementing Table (S2): List of sea bream betanodavirus isolates included in the supplementary phylogenetic analysis. Sea bream betanodavirus isolates included in the supplementary phylogenetic tree, that comprises also RNA2 sequences related to viruses for which the RNA1 sequence is not available or spans a different region of the polymerase gene from that used in the present study. The following information is reported: yeas of isolation, age of the specimen, fish status, country or farm of origin, presence of clinical signs, GenBank accession numbers for RNA1 and RNA2 sequences and reference.

ID isolate	Year	Age	Fish status	Origin	Clinical signs	GenBank Acc. No.		Genotype	Reference
						RNA1	RNA2		
PtSa-IAusc345.05	2005	Larvae	Farmed	Portugal	n.a.	GQ131549	GQ131570	RGNNV/SJNNV	1
PtSa-IAusc347.05	2005	Larvae	Farmed	Portugal	n.a.	GQ131561	GQ131571	RGNNV/SJNNV	1
592.04	2004	Larvae	Farmed	Portugal	n.a.	GQ131552	AM110730	RGNNV/SJNNV	1,2
70.05	2005	Larvae	Farmed	Portugal	n.a.	GQ131562	AM110739	RGNNV/SJNNV	1,2
PtSa-IAusc46.05	2005	Larvae	Farmed	Portugal	n.a.	GQ131559	GQ131566	RGNNV/SJNNV	1
24.05	2005	Larvae	Farmed	Portugal	n.a.	GQ131560	AM110734	RGNNV/SJNNV	1,2
72.05	2005	Larvae	Farmed	Portugal	n.a.	GQ131547	AM110740	RGNNV/SJNNV	1,2
PtSa-IAusc22.05	2005	Larvae	Farmed	Portugal	n.a.	GQ131556	GQ131565	RGNNV/SJNNV	1
PtSa-IAusc08.06	2006	Larvae	Farmed	Portugal	n.a.	GQ131557	GQ131563	RGNNV/SJNNV	1
PtSa-IAusc344.05	2005	Larvae	Farmed	Portugal	n.a.	GQ131548	GQ131568	RGNNV/SJNNV	1
PtSa-IAusc23.05	2005	Larvae	Farmed	Portugal	n.a.	GQ131558	GQ131569	RGNNV/SJNNV	1
PtSa-IAusc57.05	2005	Larvae	Farmed	Portugal	n.a.	GQ131553	GQ131567	RGNNV/SJNNV	1
45.05	2005	Larvae	Farmed	Portugal	n.a.	GQ131555	AM110735	RGNNV/SJNNV	1,2
TunB4	2009	Larvae	Farmed	Tunisia	Absent	-	DQ462317	RGNNV	3
TunB3	2009	Larvae	Farmed	Tunisia	Absent	-	DQ462318	RGNNV	3
Tun76	2009	Juvenile	Farmed	Tunisia	Present	-	DQ462320	RGNNV	3

n.a.: not available

References:

1. Olveira, J. G. *et al.* Comparative analysis of both genomic segments of betanodaviruses isolated from epizootic outbreaks in farmed fish species provides evidence for genetic reassortment. *J. Gen. Virol.* **90,** 2940–51 (2009).
2. Cutrín, J. M. et al. Emergence of pathogenic betanodaviruses belonging to the SJNNV genogroup in farmed fish species from the Iberian Peninsula. *J. Fish Dis.* **30**, 225–32 (2007).
3. Chérif, N. et al. Viral encephalopathy and retinopathy of *Dicentrarchus labrax* and *Sparus aurata* farmed in Tunisia. *Vet. Res.Commun*. **33**, 345–53 (2009).
